# The Implementation of Infant Anoesis and Adult Autonoesis in the Retrogenesis and Staging System of the Neurocognitive Disorders: A Proposal for a Multidimensional Person-Centered Model

**DOI:** 10.3390/geriatrics10010020

**Published:** 2025-02-02

**Authors:** Alessandro Pirani

**Affiliations:** Alzheimer’s Association “Francesco Mazzuca”, Via Reno Vecchio, 33, 44042 Cento, Italy; piranibosi@alice.it; Tel.: +39-3357717899

**Keywords:** anoesis, autonoesis, retrogenesis, staging, neurocognitive disorders, Alzheimer’s disease, dementia

## Abstract

**Background:** Retrogenesis is the process by which the degenerative and vascular mechanisms of dementia reverse the order of acquisition in the normal development. **Objective:** The development of memory/knowledge after birth may help to know the biopsychosocial and functional characteristics (biosphere) of the retrogenesis. **Methods:** A literature review was performed in the PubMed, Google Scholar, and Scopus databases using 43 keywords related to retrogenesis: 234 eligible records were selected. **Results:** The infantile amnesia, characterized from anoesis, was described along the infant/child’s biosphere in which the limbic system progressively develops the acquisition of the body knowledge (Anoetic Body Consciousness, AnBC). Anoesis is the infant memory state characterized by the absence of long-term memories of the many stressful/painful experiences that accompany the acquisition under the long-life voluntary control of the long-term memories fundamental for the body growth and survival (mainly chewing/swallowing and walking). At the age of 3–4 years, usually, the AnBC evolves, as a continuum, into the adulthood autonoesis with the emergence, in the child/adolescent, of the consciousness of “self” trough the development of the Episodic Autobiographic Memory (EAM) and the Autonoetic Mind Consciousness (AuMC). The development of cognition and knowledge is due to the progressive maturation of the whole limbic system and not only of the hippocampus. In the biopsychosocial retrogenesis, the EAM/AuMC vanishes progressively along the mild, moderate, and severe stages of dementia when the infant AnBC resurfaces, losing progressively the basic activities of daily living in a retrogenetic order of acquisition where the last functions to disappear are chewing/swallowing. **Conclusion**: The transition from the adult EAM-AuMC to the infant AnBC, as a continuum in the individual biosphere, adds a contribution to the assessment of the retrogenesis in dementia from a multidimensional person-centered model.

## 1. Introduction

Retrogenesis is the process by which the degenerative and vascular mechanisms of dementia [1,2], now classified as Major Neurocognitive Disorders (NCDs) [3], reverse the order of acquisition in the normal development [4,5,6,7]. Precise relationships have been scientifically documented among the cognitive, linguistic, praxic, functional, behavioral, and feeding changes in the course of Major Neurocognitive Disorders (MaNCDs) and the inverse corresponding Piaget’s developmental sequences [8], especially in Alzheimer’s Disease (AD) [9]. Similar inverse relationships between AD and human development can be described for physiologic measures of electroencephalographic activity, brain glucose metabolism, and developmental neurologic reflex changes [10,11]. The development of a person gradually progresses from infancy and continues along the heterogeneous pathways of individual Biopsychosocial (BPS) development (biosphere) [12,13].

In particular in the hierarchical development of the biosphere from birth [14], the nervous system is the apex system that connects and manages as a whole unicum the psychological features and intellective activities that each person accomplishes in every moment of his life [15], also known as “MindBrain” [16].

As Hering wrote [17]: “Memory connects innumerable single phenomena into a whole, and just as the body would be scattered like dust in countless atoms if the attraction of matter did not hold it together so consciousness—without the connecting power of memory—would fall apart in as many fragments as it contains moments”. As a consequence, the BPS retrogenesis may be key to understand the global rewind of a person affected by MaNCDs (PwMaNCDs).

The BPS retrogenesis of the PwNCDs and its related functional stages [18] reverse into corresponding Developmental Age Equivalents (DAEs) [4]. As a fact, the developmental process of the MaNCDs replicates the ontogenetic sequence in which the neurologic structures [1], particularly the limbic system [19], evolved from infancy. The neuropathologic sequence of brain changes in AD [20] develops in the reverse order of the normal development of the cortical regions after birth [21,22,23,24,25,26,27].

The retrogenesis-DAEs have been the cornerstone pattern to understand the heterogeneity [27] of cognitive, behavioral, and functional changes of the MaNCDs and, as a consequence, to develop the staging tools to map the related needs of general care in PwMaNCDs like the Global Deterioration Scale (GDS) [28], the Functional Assessment Staging Tool (FAST) [29,30,31] and the Clinical Dementia Rating scale (CDR) [32,33].

The staging of MaNCDs is the first of ten pivotal measures to improve the needed quality in the holistic management of PwNCDs [34].

Basically, the BPS retrogenesis must be amended by necessary caveats, common to all the PwMaNCDs, regarding the multidimensional assessment [35,36,37] of physical, comorbid, and societal differences and, in the background, between PwMaNCDs and their developmental age “peers” [2].

More specifically, the BPS retrogenesis takes into account the intrinsic psychic–intellectual capacity [creativity, Intelligence Quotes (IQ), self-learning/education, motivation toward life, environment, openness to experience] [38,39] with which every person develops and mixes from infancy the six domains of cognition, namely: complex attention, executive function, learning and memory, language, perceptual–motor function, and social cognition with relative sub-domains [40].

In particular, the domain of socio-emotional cognition explains how socially inappropriate behaviors, usually termed as “behavioral and psychiatric symptoms of dementia” (BPSD) [41,42] and recently implemented as “agitation” [43], can manifest as a disruptive feature in 60–75% of PwMaNCDs [44,45]. The socially inappropriate behaviors and related symptoms are caused by the progressive loss of the acquired ability (autonoesis) to inhibit unwanted behaviors, recognize social cues, read facial expressions, express empathy, motivate oneself, alter behavior in response to feedback, or develop insight [1,40].

Therefore, in the retrogenesis pathway, one relevant issue is the reversal from autonoesis, the autobiographical and behavioral awareness arising at 3–5 years of age [46,47,48], to anoetic consciousness, best known as infantile amnesia (IA) [49,50]. The transition of socio-emotional cognition from infancy into adolescence and adulthood is characterized by the progressive amelioration of cognitive control and decision-making that are dependent from specific cognitive capacities co-evolving with complex network effects within and between different “Resting-State Networks” (RSNs), throughout postnatal development [51]. As a consequence, the progressive loss of autonoesis in PwMaNCDs is due to the related continuous impairment of the RSNs from the level of development that the six domains of cognition reached in the adult brain’s functional organization [52,53].

The BPS retrogenetic transition from autonoesis to IA adds one more caveat regarding the corresponding DAEs in the “moderately severe/profound” and “severe/terminal” stages of the MaNCDs: they may elapse until 20 years before death and are characterized with the most challenging issues for the caregivers and physicians as regards the care and management of PwMaNCDs.

As a consequence, the acquaintance of the development of memory/knowledge after birth and in childhood–adolescence [54,55,56,57] is a relevant issue for physicians and caregivers to understand the retrogenesis of the PwMaNCDs biosphere to which parallelly adapt caring [58].

For this purpose, the aims of this work are: (1) to detail, briefly, the development of memory/knowledge after birth and its physiological transition from IA to autonoesis; (2) to describe the BPS and functional characteristics of the retrogenesis from autonoesis to IA in PwMaNCDs; (3) to unify the cognitive, behavioral, and functional changes of MaNCDs in the etiopathogenetic involvement of the individual biosphere; (4) to simplify the learning of the prognostic staging of PwMaNCDs developing visual models of retrogenetic BPS person-centered impairment and care useful for the non-specialists in the field physicians, mainly general practitioners, nurses, and caregivers.

## 2. Methods

The literature review was performed by consulting the PubMed, Google Scholar, and Scopus databases, all of which were consulted from January to November 2024, with unlimited searches as regards years. The inclusion criteria were: English language, literature reviews, meta-analyses, primary research studies, books. A phrase search was performed with the individual terms separated by the AND operator.

The research was conducted using a wide range of retrogenesis-related keywords for each domain.

The 43 keywords used were: infantile amnesia, episodic memory, ontogeny of memory, autonoesis, anoesis, consciousness, neurocircuitry of memory, amygdala, nucleus basalis of Meynert, hippocampus, emotion, cognition, biopsychosocial model, major neurocognitive disorders, mild neurocognitive disorders, dementia, Alzheimer’s disease, frontotemporal dementia, neuropathological staging system for NFTs/NTs staging of AD, biological clock, circadian rhythm, non-pharmacological treatment, hypothalamic–pituitary–adrenal-gonadal axis, behavioral and psychiatric symptoms of dementia, agitation, advanced activities of daily living, instrumental activities of daily living, activities of daily living, swallowing solids, balance and gait/walk, fear, fear of falling, sleep–wake rhythm, multidimensional comprehensive geriatric assessment, amyloid–tau–neurodegeneration, neurofibrillary tangles, brain cholinergic denervation, cognitive stimulation, reality orientation therapy, elder-clowning, pet therapy, doll therapy, staging tools for dementia.

The abstract of each record was analyzed to determine its relevance as regards the knowledge of the BPS and functional characteristics of how memory works during the three structural topics:

(a) Infancy;
(b) Childhood–adolescence–adulthood;
(c) Retrogenesis.

## 3. Results

The following results were obtained on the basis of: (a) primary research studies: *n* = 86, (b) original articles: *n* = 41, (c) reviews: *n* = 26; (d) meta-analysis: *n* = 5, (e) anatomic articles: *n* = 19, (f) books: *n* = 15.

### 3.1. Infantile Amnesia

In the ontogeny of memory, IA refers to the absence of Episodic Autobiographical Memory (EAM) [59] in the first 3–5 years of life and, after then, the first few EAMs begin to consolidate, marking the beginning of metamemory [60,61] at approximately 4 to 7–8 years of age [62]. Freud is acknowledged as the first scientist who defined IA as the “failure of memory for the first few years of life”, underlining the emotive and psychological impact that these experiences can have throughout the lifespan [63].

The IA is present in all the other mammalian brains, to different extents [64,65].

In addition to IA, the infant’s memory is characterized by other relevant phenomena like Infantile Generalization (IG), the bias toward memory generalization during early development, the enhanced extinction and reversal learning, and the temporary suppression of fear memories [62,66,67,68].

The research argues if IA is due to a failure of memory storage or memory retrieval [69]. Nevertheless, this approach considers the infant’s memory, characterized by the absence of EAM, to be shaped like the adolescence–adulthood one, where, instead, the growing functioning of EAM is the basis for the development of the “self” after and not together with IA [59].

In fact, it has been demonstrated that the hippocampus, the basic structure of memory [70,71], starts to develop and learn after birth [72,73], but the inputs [74] and outputs [75] that the brain must govern during the IA are totally different from the person’s ones, slowly emerging after IA until adulthood. In infancy, the inputs are related to the specific needs “quoad vitam” of the first period of life and the outputs are connected to the progressive discovery, control, and conquering of the body coupled with the ongoing hippocampal and brain maturation [15].

The acquisition and governing of the body are the first natural and essential steps [76] for switching from “mother and nest dependency” toward gaining the ultimate and completely different goal that is the development of autobiography and the control of “me/self” [77,78], in a word, the achievement of independence. The development of “me/self” is related to the never-ending maturation of socio-behavioral cognition (“quoad valetudinem”) from childhood until longevity [79,80] that drives the individual’s rapid development of other cognitive domains from childhood [81,82,83,84,85].

**Figure 1:**
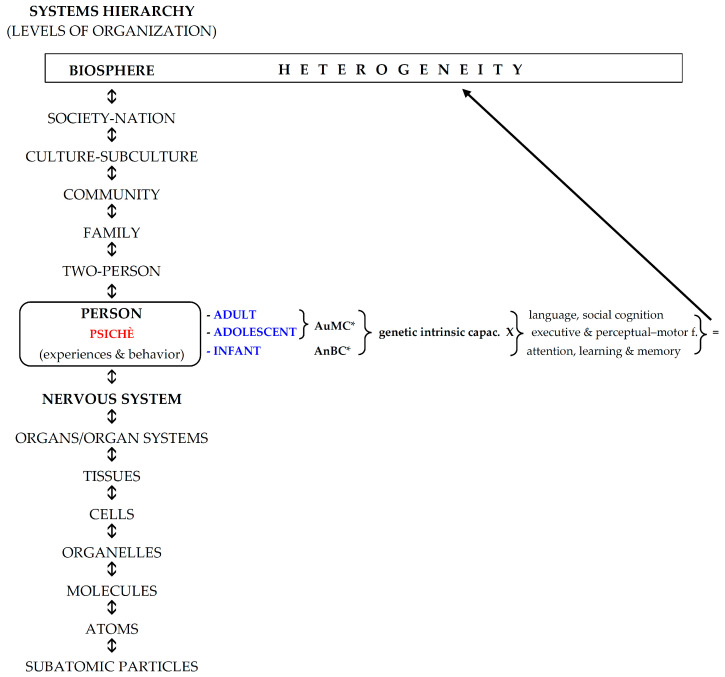
The hierarchy of natural systems: pathway of the development of the individual Biopsychosocial (BPS) sphere, adapted from [12]. AnBC*: Anoetic Body Consciousness; AuMC*: Autonoetic Mind Consciousness.

**Figure 2:**
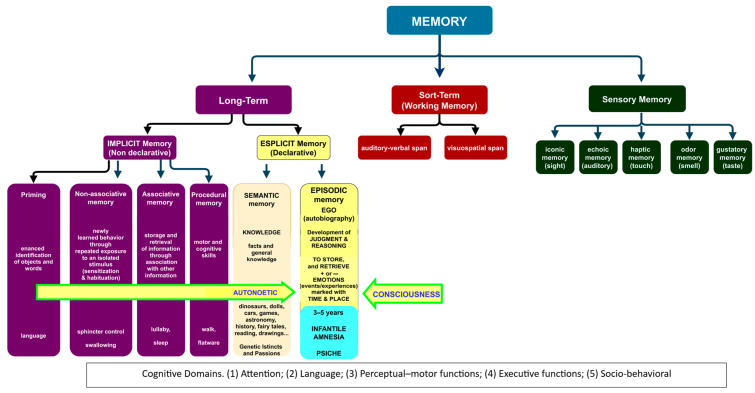
A proposal of the functioning of memory from birth to adulthood integrated in the other five cognitive domains, adapted from [90].

Therefore, the absence of EAM in infancy should not be a wonder, and the question is: why cannot the EAM develop in infancy? A suggestion to answer this question will be attempted inserting the development of the newborn as an ecological whole entity from an evolutionary BPS and functional perspective [86]. In this light, the original BPS pathway should be implemented in the central “person” step by adding the “psichè”, the immaterial, genetic engine of the person that steers the three developmental seasons of life: infancy, adolescence, and adulthood (young, middle, and longevous) (Figure 1). The reasons for this approach are the different psychological inputs and the related outcomes (experiences, functions, behaviors) that the six basic domains of cognition (complex attention, executive function, learning and memory, language, perceptual–motor function, and social cognition) process in the different seasons of life [87] shaped from the different socio-cultural ethnicities [88] (Figure 1). In fact, if we consider the life development from the point of view of the absence/presence of EAM, the “person” step of the BPS pathway can be rearranged in two genetic developmental stages to which the “nervous system” concurs in the same way (knowledge) but with different strategies and outcomes: infancy and adulthood. In infancy, the knowledge is characterized by the progressive attainment of the anoetic consciousness of the body (anoetic body consciousness: AnBC), at the end of which starts the transition to adulthood, via the adolescence transitional period, characterized by the progressive development and growth of the autonoetic consciousness of the own mind (autonoetic mind consciousness: AuMC, “cogito ergo sum”) [89] (Figure 2).

As a consequence, the analysis of the BPS pathway of the two genetic developmental stages is re-arranged in their ontogenetic roles, rather than limited to their physiological organ supports, as follows: first, the socio-functional goals, second, the psychological strategies (developmental psychology), and, third, the related biological domain (neurobiology and neurocircuitry).

### 3.2. The Development of Knowledge in Infancy

#### 3.2.1. Genetic Socio-Functional Goals: To Conquer the Body

In humans and the other warm-blooded species (mammals and birds), the infancy is the pivotal basic step needed to become adult. The genetically inherited aim of every newborn is to achieve and conquer the complete control of his own body in its ecological niche [91,92]. The socio-functional aims of the human infant knowledge are the attainment of the basic functions pertaining the independence of the body (Activities of Daily Living—ADL) [93] (Table 1). These functions are progressively gained along with the development of the body integrated with the organs and systems (brain, sense organs, muscles, bones, primal teeth, …) [94].

#### 3.2.2. Psychological Infantile Strategies: Background, Inputs/Outputs

Environmental social influences, especially prenatal and early postnatal life experiences with the mother, father, and other caregivers, prepare the developing neural and neuroendocrine systems [95], which have been organized in the embryo and fetus [96], for adaptive reactions to the demands and stresses of a young person’s life [97,98]. Brain organization is revised and updated through processes of meeting complexities of different systems or moment-to-moment meetings between the caregiver and the infant [99].

The children could not permanently judge what originates from personal experience before the age of 3, because, in this stage, brain development is the product of a complex series of dynamic and adaptive processes operating within a highly constrained, genetically organized but constantly changing context, leading to the advent of metamemory–metacognition [61,100].

It can be argued that the development of language and semantic memory during the AnBC is an interactive, strategic process that permits the birth of new and different knowledge linked to time–space and, mainly, to a changing emotional individual context, leading to the rise of the permanent memories of AuMC/EAM.

The development of language permits the infant to substitute the monomodal vocal alarm signals, like crying, with which to catch the mother’s attention, with the multiform verbal interaction for discovering, understanding, and fixing the surrounding world.

So, the AnBC stage is polarized to acquire and stock the long-term memories relative to body control (Table 1) that merge to the subsequent stage of AuMC.

The absence of permanent judgment in AnBC is strategic in the infant’s experiences for the acquisition of three functions of the body: hygiene, swallowing solids, and balance and gait/walk. In fact, these experiences may be very unpleasant, like the shocking abandonment of the warm breastfeeding, forcing the mouth with unpalatable things to be swallowed in a new way, or the fearful acquisition of balance and the many painful falls that lead to the gait/walk control.

It is evident that the infant AnBC considers these frequent, unpleasant, and painful episodes [101] as temporary inconveniences that are rapidly forgotten because not stored as “threats/dangers” like it would happen in a frontally controlled adult. If the infant had a developed AuMC, these troubles would be classified as threats/dangers for life and he would never learn to swallow solids or to gain balance and gait/walk.

The Fear of Falling (FOF), in the longevous frail but not demented adult, represents reverse evidence of this behavior. FOF is an important public health problem, causing excess disability among older people [102]. FOF begins with poor confidence in mobility because of a fall or other physical problems affecting gait and balance. The advent of FOF is due to the good functioning of the longevous adult’s AuMC/EAM, from which the concept of threats/dangers consequent to a fall (always pain, sometimes fractures) is retrieved, leading to a self-imposed restriction of activity and an increasingly sedentary lifestyle [103].

The development of EAM is greatly influenced by the maternal relationship: the way in which the mothers assemble their conversations when reminiscing with their children has a relevant impact on the inception of EAM and the way with which the children describe their past. The maternal reminiscing style is, however, moderately mediated by the nature of the mother–child affection that, when insecure, may negatively impact on the quality of the kids’ EAM [59].

The research considers four hierarchal models of self-development [59]: firstly, a very basic “proto-self” grounded in the sensory and motor domains, secondly, a pre-linguistic, affective “core self”, followed by a semantic “cognitive self” and, then, by an “autobiographical-narrative self” (EAM).

In infancy, this process is mediated by a primal system that comprehends four categories of inborn affects: “instinctive, emotional, homeostatic and sensory” (Table 2) [99,104].

In the light of the retrogenesis and DAE, the affects, particularly the emotional and sensory ones (Table 3), appear to be variously related to the resurfacing of the toddler/child’s temperament and behaviors in PwMaNCDs, but with the difference that, in toddlers/children, the affects are finalized to their homeostasis, [105,106] while they become non-finalistic in PwMaNCDs [107]. In fact, the progression of DNC to advanced stages like “moderately severe” and “severe” in GDS/FAST or “profound” and “terminal” in CDR [33] brings the patients to lose the prefrontally driven personal and social meanings of the acquired ADL (see: 3.2.1) and, consequently, PwMaNCDs’ behaviors may become problematic, oppositive, and dangerous for the caregivers [1] due the weight and strength of their adult body. Moreover, PwMaNCDs recover the infant sensory instinct like the sweet taste or emotional states like uncontrolled fear [108] or playing [109].

The resurfacing of non-finalistic behaviors in PwMaNCDs are defined as “Behavioral and Psychiatric Symptoms of Dementia” (BPSD) [41,110,111] and “Agitation” [43].

#### 3.2.3. Neurobiology and Neurocircuitry

Human developmental studies emphasize that children first acquire the semantic memory system and only thereafter their EAM neurocognitive capacities emerge [59].

**Figure 3:**
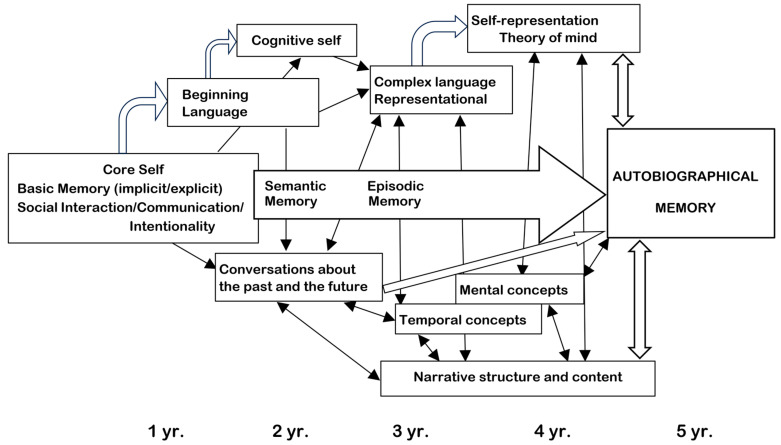
A proposal of the functioning of memory from 1 to 5 years of age, leading to the emergence of episodic autobiographical memory. Reproduced with permission from [87] (Clearance Center’s Rights Link Order Number 5943240510878).

In fact, the development of semantic memory, in concert with language [112] and conceptual knowledge, is essential to acquire adult knowledge, the ability to “understand and explain” (Figure 3).

This ability progressively substitutes the indistinct on–off “crying–smiling” parental bond and primal system of communication (Table 2) with higher levels of self and self-understanding, of understanding the feelings and intentions of others, of executive functions and working memory (Figure 2 and Figure 3), acquiring the capacity for mental time traveling and the maturation of the nervous system [59,113].

As regards the development of language, the on–off “crying-smiling” primal system, vital for survival, may be resembled to the afterbirth breastfeeding stage, before the acquisition of swallowing solids as regards the development of swallowing.

These core affects are elaborated in ancient subcortical networks that exist in all mammalian brains [114], integrated with hippocampal learning [72,115]. It is well-known that the progressive AnBC is characterized by the acquisition of many long-term bodily memories (swallowing, walking, sphincteric control, etc.) whose development occurs under the voluntary systems already active after birth. In fact, we can reject a bad-tasting food and contrast an imbalance while walking both when infants and adults. So, it can be argued that both AnBC and AuMC, even if targeted by the different aims/skills of infancy and adulthood, develop long-term memories as a continuum [116].

The research has cleared that the differences in long-term memory of AnBC versus AuMC are explained by the specific patterns of differential anterior and posterior functional connectivity of the hippocampus with an increase in the functional specialization along the long axis of the hippocampus and a dynamic shift in hippocampal connectivity patterns that supports memory development [98].

**Figure 4:**
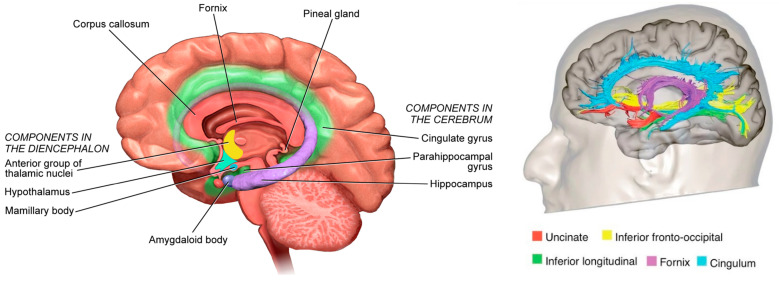
The limbic system.

Braak detailed the functional anatomy of memory from infancy that constitutes of the limbic system (Figure 4) [19]. The somatosensory, visual, and auditory inputs proceed through neocortical core and belt fields to a variety of association areas, and, from here, the data are transported via long corticocortical pathways to the extended prefrontal association cortex.

**Figure 5:**
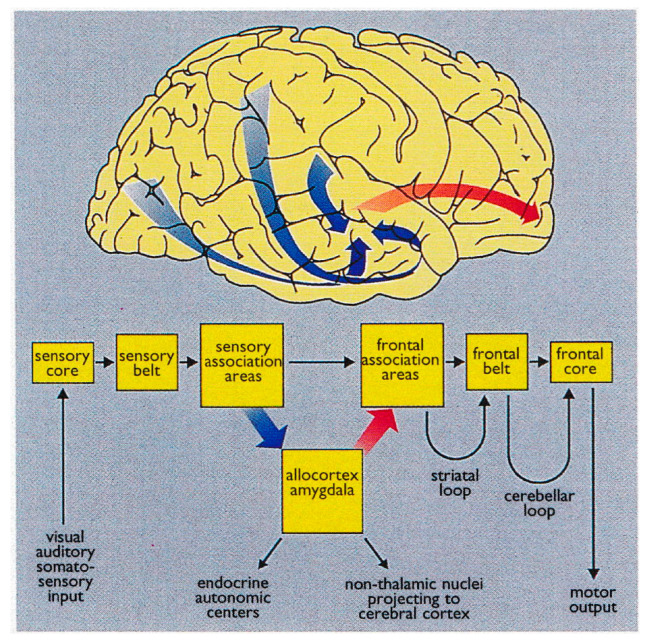
The limbic loop. The afferent leg (blue arrow) from the sensory association areas to the prefrontal cortex branches off to eventually converge upon the entorhinal cortex and amygdala. The efferent leg (red arrow) from the entorhinal region, amygdala, and hippocampal formation influences the prefrontal cortex. Reproduced with permission from [120].

**Figure 6:**
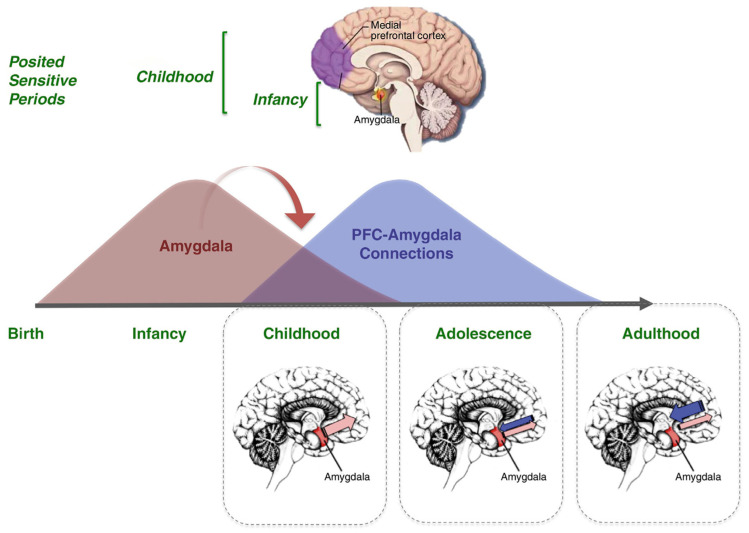
The amygdala in the different developmental ages: transition from infancy to childhood and progressive integration with the Prefrontal Cortex (PFC) along with adolescence and adulthood. Reproduced with permission from [119] (Clearance Center’s Rights Link Elsevier Order Number 5898850421278).

Tracts generated from this highest organizational level of the brain guide the data via the premotor cortex (frontal belt) to the primary motor area (frontal core). The striatal and cerebellar loops provide the major routes for this data transfer (Figure 5). The main components of the limbic system (the hippocampal formation, the entorhinal region, and the amygdala) maintain a strategic position between the sensory and the motor association areas. Part of the stream of data from the sensory association areas to the prefrontal cortex branches off and eventually converges on the entorhinal region and the amygdala [117,118,119] (Figure 6).

These connections establish the afferent leg of the limbic loop. In addition, the limbic centers receive substantial input from nuclei processing viscerosensory information. The entorhinal region, the hippocampal formation, and the amygdala are densely interconnected. Important among these connections is the perforant path, which originates in the entorhinal cortex and projects to the hippocampal formation (fascia dentata, Ammon’s horn, and subiculum) (Figure 5).

The subiculum projects to the amygdala, entorhinal region, mamillary nuclei, and anterior and midline thalamic nuclei. The hippocampal formation, the entorhinal region, and the amygdala generate the efferent leg of the limbic loop which is directed toward the prefrontal cortex (Figure 5) [121]. Additional projections reach the key nuclei that control endocrine and autonomic functions: the Hypothalamic–Pituitary–Adrenal-Gonadal (HTPAG) axis. The HTPAG axis is a complex system of neuroendocrine pathways and feedback loops that function to maintain physiological homeostasis [95]. Furthermore, the amygdala exerts influence on all nonthalamic nuclei projecting in a nonspecific manner to the cerebral cortex (i.e., the cholinergic magnocellular forebrain nuclei, the histaminergic tuberomamillary nucleus, the dopaminergic nuclei of the ventral tegmentum, the serotonergic anterior raphe nuclei, and the noradrenergic locus coeruleus) [122].

The limbic loop centers perform the integration of exteroceptive sensory data from various sources with interoceptive stimuli from autonomic centers. Their efferent projections exert influence on both the prefrontal association cortex and the key centers controlling endocrine and autonomic functions [19].

The advent of time awareness, time orienting, and proper handling of time epochs and temporal order (mental travel) are related especially to the after-birth development of the prefrontal and parietal cortices. The absence of time encoding in the first years of infancy is the essential physiological and neurobehavioral reason that explains the related absence of EAM [59] and circadian rhythms [123].

Another essential reason that explains the absence of EAM in infancy is related to the absence of a developed gender-oriented sexuality. It is known that the HTPAG axis secrets gonadal hormones in the post-natal period, but the concentrations are low and address only the development of the future sexual behavior [124,125,126,127].

Therefore, the absence of EAM and sexuality permits the infants, independently from the gender, to look at the breast as a warmful and pleasant tool for eating and not as a sexually attractive zone as in adolescence/adulthood.

The long-term memories acquired during the development of the AnBC remain stocked in a deep fundamental nucleus, in a word, like a building basement, over which, as a continuum, after the 3–5 years of age, the intertwined blocks of the AuMC are progressively placed to build the “pyramid” of the long-life knowledge.

### 3.3. The Development of Knowledge in Childhood–Adolescence–Adulthood

#### 3.3.1. Genetic Socio-Functional Goals: To Conquer the Mind/World

The progressive development of knowledge in infants after 3–4 years of age pointed to autonomy shapes the transition from anoetic consciousness or IA/AnBC to the advent of autonoetic awareness or EAM/AuMC [59,128].

**Figure 7:**
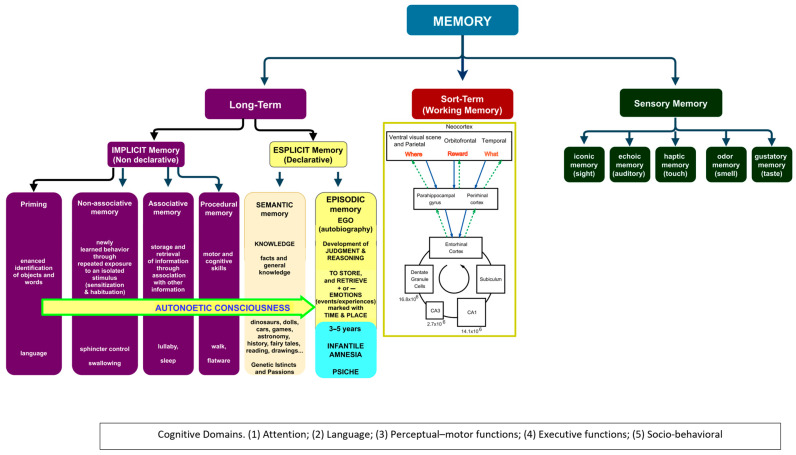
The emergence of autonoetic consciousness/episodic memory from anoetic consciousness (infantile amnesia), adapted from [71].

The turning point is usually at the age of 3 years when the infant begins to walk upstairs with alternating feet, to join in make-believe play with other children, and to ask adults “what?”, “where?”, “who?”, “when?” and “why?” [129], merging the answers with “reward” and storing them as “mental time travel”, thus developing the EAM/AuMC (Figure 7) [71].

After the infant AnBC, the autonoetic childhood–adolescence is committed to acquire two parallel genetic targets that are independence and sexuality, both pivotal to reproduction, the foundational life’s goal.

As regards independence, it develops during childhood and adolescence, acquiring the knowledge of the intermediate socio-functional states of autonomy, largely overlapping with the Instrumental Activities of Daily Living (IADL) of the longeval adults (https://www.alz.org/careplanning/downloads/lawton-iadl.pdf, accessed on 18 November 2024) [130].

As regards sexuality, the developmental tasks of childhood–adolescence comprise accepting one’s body, adopting a gendered social role, achieving emotional independence from the parents, developing close relationships with peers of the same and opposite gender, preparing for an occupation, preparing for marriage and family life, establishing a personal value or ethical system, and adopting socially responsible behavior (Figure 1). Thus, adolescent development largely focuses on issues related to sexuality [125].

In turn, the adolescent autonomy is the necessary stage to achieve the (legal) adult independence: the right to vote, the ability to work (complex activities: advanced activities of daily living) [131,132], the responsibility to get married and generate sons [133,134,135].

#### 3.3.2. Psychological Strategies: Background, Inputs/Outputs

The infant state of AnBC progresses through brain states from which capacities for higher forms of consciousness gradually emerge, with brain–mind encephalization leading to EAM/AuMC [136]. The EAM is “...a past- and future-oriented, context embedded neurocognitive memory system that receives and stores information about temporally dated episodes or events, and temporal-spatial relations among them from one’s past” [137,138].

The consolidation and recall of these EAMs from one’s past are regulated by states of anoetic affective consciousness. Consequently, implicit self-relevance becomes intimately related to each individual’s biosphere that encompasses unique feelings, thoughts, goals, and behaviors.

The EAM differs from other forms of memory (Figure 7) because it requires an extended sense of self that engages in mental time travel: it marks past events generating future scenarios and options [47].

The self, AuMC, and EAM are inputs strictly interlocked, both ontogenetically and phylogenetically, and overlap with each other, enhancing their mutual development. Their appearance and development take place in concert with other cognitive and emotive functions—the ability of perspective taking, executive functions, language, the feeling of empathy, the ability to reflect on oneself, solving abilities with divert thinking, etc. [139].

**Figure 8:**
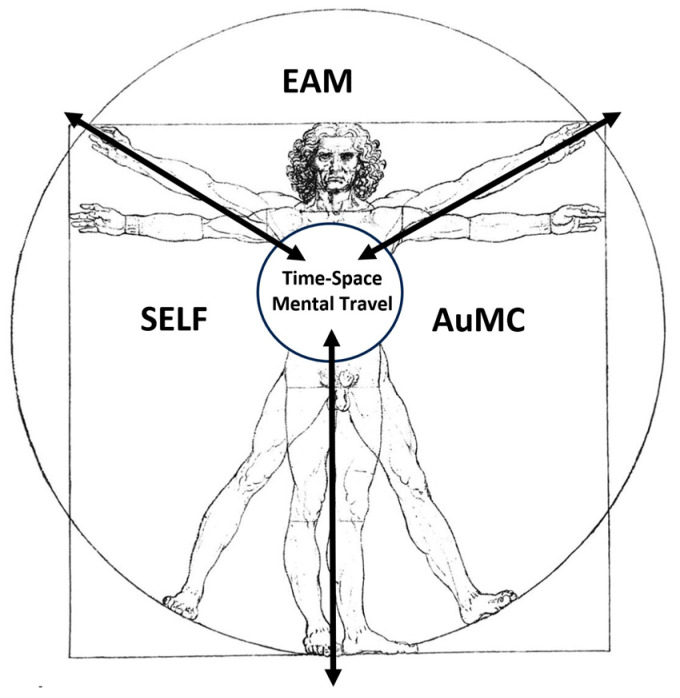
The multiplicity/multimodality of the self: a perspective of the overlapping relationships of Episodic Autobiographical Memory (EAM/AuMC) and self and their embeddedness in time–space and mental travel (EAM), adapted from [59]. EAM: Episodic-Autobiographic Memory; AuMC: Autonoetic Mind Consciousness.

The relationship among self, AuMC, and EAM can be represented in a Vitruvian man silhouette where the “multiplicity/multimodality of the self of the EAMs” (semantic representations of one’s personality traits, semantic knowledge of facts about one’s life, experience of continuity through time, sense of personal agency and ownership, ability to self-reflect, and the physical self) [140] represent inputs that lead to the individual ecological, interpersonal, conceptual, remembered, and private self outputs (Figure 8) [141].

#### 3.3.3. Neurobiology and Neurocircuitry

AnBC is a state of pre-reflective affective and sensorial perceptual consciousness essential for the waking state of the organism in the absence of an explicit self-referential awareness of associated cognitive contents. This state of affective and sensorial consciousness acts as a bridge that connects deeply unconscious information processing, primordial affective feelings, and perceptual consciousness with the possibility of knowing (noetic) levels of consciousness situated within the basal ganglia (amygdala [122], nucleus accumbens, bed nuclei of the stria terminalis), which mediates learning and memory, and higher regions of the brain, such as the neocortex, which allows for mental time travel from the permutations of these memories (EAM/AuMC) [136].

Later in the development, when reflection is possible, those primal affects act as free-flowing streams underlying the more cognitively detailed aspects of our continuously ongoing cognitive–noetic and autonoetic information processing as thoughts, images, fantasies, expectations, and anticipations. In contrast to anoetic consciousness, autonoetic consciousness refers to the reflective capacity to mentally represent a continuing existence that is embedded in specific episodic contexts and associated with remembered experiences with an affective quality—from “warmth and intimacy” to “dread and alienation” [136].

The functions of self, AuMC, and EAM (Figure 8) are mediated from portions of the prefrontal cortex, in particular its ventromedial and lateral right hemispheric regions. The anterior and posterior cingulate cortex, the precuneus, and the temporo-parietal junction area are also engaged, in addition to the medial prefrontal regions [59].

Time awareness, time orienting, and a proper handling of time epochs and temporal order are related especially to the prefrontal cortex, while the proper perception of time and time epochs seems to engage the parietal cortex as well as the diencephalic structures, as mentioned before [59].

The ability to re-experience the past events and pre-experience personal future events might also be severely impaired after bilateral medial temporal lobe damage [97].

The personal ownership of mentalizing, probably most developed in humans, is deeply mediated by the hippocampi and frontal lobes evolution and microstructure. The neural correlates of EAM/AuMC are linked to various memory abilities, especially declarative memory, and AnBC is strongly connected to raw sensorial and perceptual abilities, various subcortical affective processes, and intrinsic affective value structures. These processes take place into the limbic and paralimbic structures associated intrinsically with the more implicit free flow of affective consciousness [136].

The emergence of EAM/AuMC in childhood is coupled with the progressive stabilization of the biological clock, adapted to the light–dark cycle, that regulates the circadian rhythms for sleep–wake, hormone production, and, importantly, memory [142].

The neural system of the biological clock is the paired Suprachiasmatic Nuclei (SCN) in the anterior hypothalamus, located above the optic chiasm at the base of the third ventricle. The SCN has input and output pathways, i.e., with the HTPAG axis, and exhibits endogenous rhythmicity with a period of oscillation close to 24 h [142].

The SCN controls output timing with respect to peripheral oscillators, i.e., the liver, heart, and discrete regions of the brain, such as the hippocampus, to coordinate the daily cycling of numerous essential physiological processes [123].

The stabilization of the biological clock, including the times for eating and sphincteric control, allows the development of progressively organized daily activities [123].

One foundational element that influences EAM/AuMC as regards the progress of social cognition and related behaviors is the development of sexuality due to the maturation of the HTPAG axis [143,144,145,146].

Sexuality and developmental control behavior are based on a critical equilibrium between personal expectations and desires and partner’s fulfillment, whose disappointment may cause sexual desire discrepancy and frustration. Sexual frustration describes a state of irritation, agitation, or stress resulting from sexual inactivity or dissatisfaction. Sexual frustration is a common, natural feeling and it can affect anyone. Sexual frustration is a natural response that many people experience at one time or another, but, not rarely, it may cause a state of irritation, agitation, or stress or degenerate in aggressiveness and violence [147,148,149,150,151,152].

### 3.4. The Biopsychosocial and Functional Characteristics of the Retrogenesis

**Figure 9:**
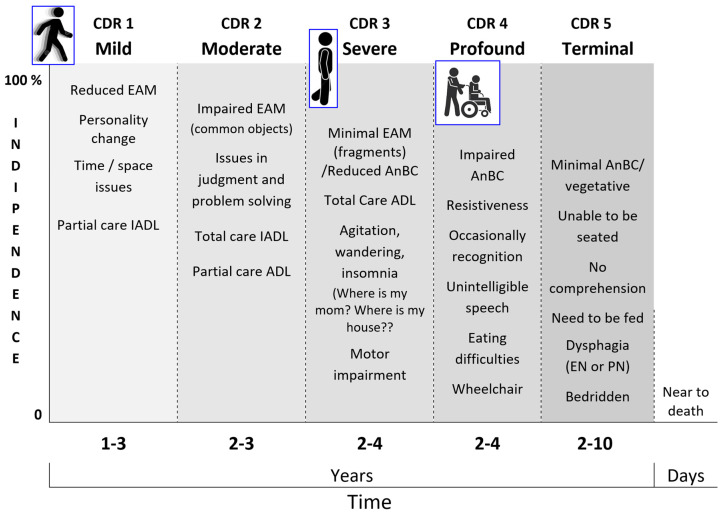
A synopsis of the biopsychosocial and functional cascade of neurocognitive disorder corresponding to the stages of CDR (stages 0-normal and 0,5-questionable were not included). CDR: Clinical Dementia Rating extended [32,33]; EAM: Episodic Autobiographical Memory; AnBC: Anoetic Body Consciousness; IADL: Instrumental Activities of Daily Living; ADL: Activities of Daily Living; EN: Enteral Nutrition; PN: Parenteral Nutrition.

The development of a person from birth and his encapsulated Central Nervous System (CNS) is a pathway to assess PwMaNCDs in their comprehensive BPS and functional retrogenetic cascade (Figure 9). This approach points to examine PwMaNCDs with the methodology of multidimensional comprehensive geriatric assessment [36,153] to unify the assessment of the BPS and functional domains of the person as a whole and a continuum and not simply the illness and its manifestation [18].

The long-term memories of the infant/child AnBC, apparently closed forever in the “basement” under the “building” of the AuMC, progressively resurface during the development of MaNCDs, from severe until the terminal stages (Figure 9). As for the retrogenesis of AuMC, the resurfacing of AnBC occurs with the same modalities, manifesting, initially, as the loss of the last functions acquired in AnBC, like sphincteric control in the severe stage, and proceeding until the frequent loss of swallowing in the after birth—vegetative terminal stage (Figure 9).

**Figure 10:**
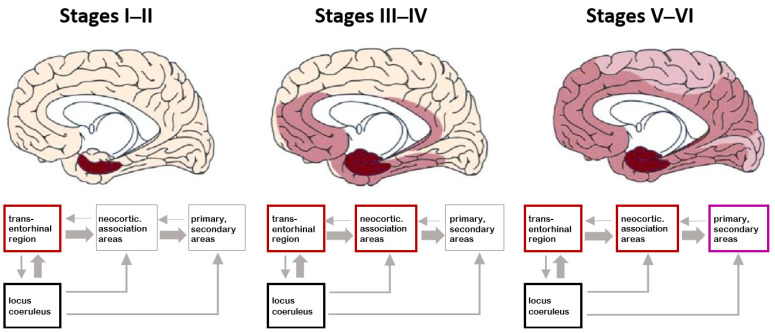
The neuropathological staging system for NFTs/NTs proposed by Braak in the development of Alzheimer’s disease. Reproduced with permission from [156].

Braaks’ s work was pioneering not only in detailing the anatomy and functioning of the limbic system related to the emergence of EAM/AuMC, but, parallelly, in creating the AD neuropathological staging system for neurofibrillary tangles (NFTs) and neuropil threads (NTs), a six-stages tool based on the progressive accumulation of NFTs in the limbic system (Figure 10) [115,154,155,156,157,158].

**Figure 11:**
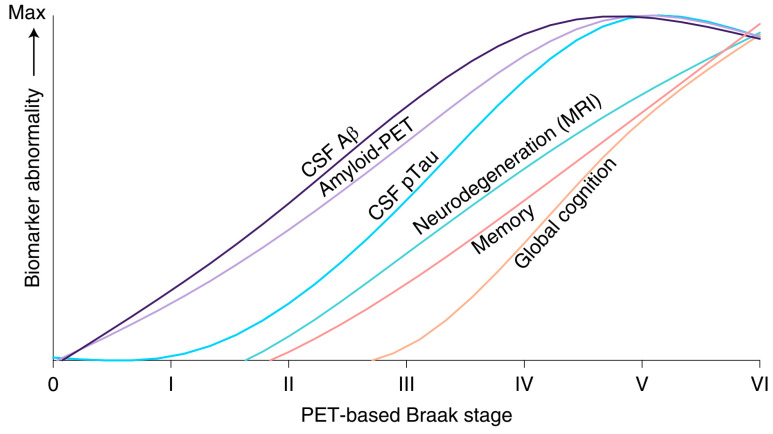
Correlation between the neuropathological staging system for NFTs/NTs proposed by Braak and the abnormalities of the biomarker in Alzheimer’s disease [161].

The neuropathological staging protocol for NFTs/NTs proposed by Braak [157] is a pivotal marker of BPS retrogenesis [115,155] whose appropriateness was recently confirmed with the cognitive [159,160], staging [32], and new neuroimaging biomarkers now available (Figure 11) [161].

**Figure 12:**
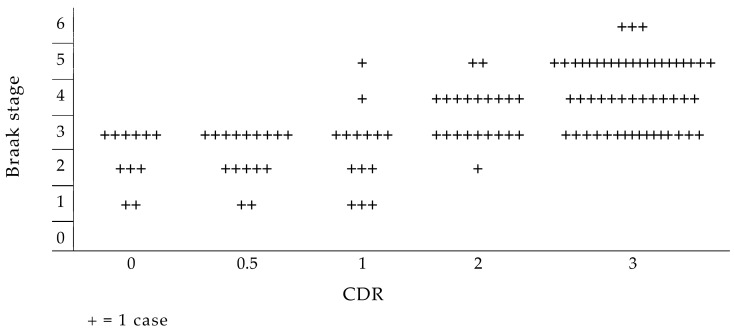
Correlation between the neuropathological staging system for NFTs/NTs proposed by Braak and the CDR. Reproduced with permission from [162]. (Clearance Center’s Rights Link Order Number 5956541377490).

In details, the neuropathological staging system for NFTs/NTs proposed by Braak in the development of Alzheimer’s disease is strictly correlated with the worsening of CDR (Figure 12) [162,163,164].

#### 3.4.1. The Biological Domain

MaNCDs affect the limbic system and the other connected regions with different etiopathogenesis (amyloid–tau–neurodegeneration, cerebrovascular disease, Lewy bodies…) and modalities responsible for the different clinical forms like Alzheimer’s, frontotemporal, vascular, Lewy body, and mixed [165].

The retrogenetic cognitive, behavioral, and functional cascade is common to all MaNCDs, but its clinical manifestations, mainly in the mild and moderate stages of CDR, may change due to different etiopathogenetic involvements of the limbic system [9]. Damage to the parietal cortex as well as the diencephalic structures may disturb the sense of time considerably (including the ability to successively link events in time) [166] (Table 4).

As a consequence, PwMaNCDs become unable to encode or store new EAMs successfully because the capacity for episodic future thinking (self-projection in the future) is an essential feature of EAM/AuMC. Severe impairment of EAM has been found in patients with damages to the amygdaloid complexes and related circuitry with forebrain regions [168].

**Figure 13:**
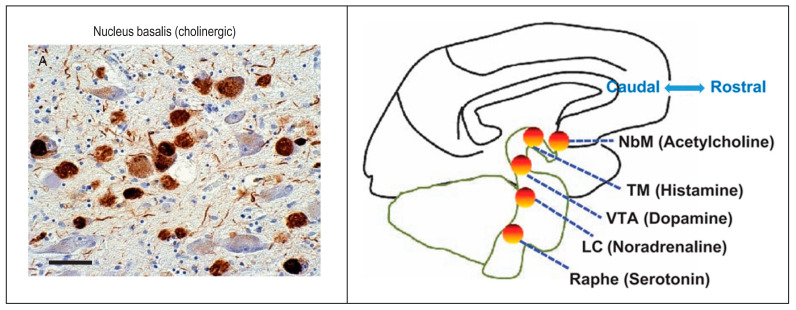
Neurofibrillary Tangles (NFTs) in Ascending Reticular Activating System (ARAS) including the acetylcholinergic Nucleus basalis of Meynert (NbM) and the other ARAS nuclei with their neurotransmitters (Tuberomammillary Nucleus (TM), Locus Caeruleus (LC), Ventral Tegmental Area (VTA), brainstem raphe). Reproduced with permission from [169] (Clearance Center’s Rights Link Order Number 5943241226668).

A special anatomical consideration referent to NFTs in AD is the impact of NFTs on the Ascending Reticular Activating System (ARAS) [169]. ARAS neurons send strong inputs to the cerebral cortex that augment memory, arousal, attention, and motivation. These cells are also severely affected in AD. For example, considering the brain cholinergic denervation [170], the impact of NFTs on the ARAS cholinergic Nucleus basalis of Meynert (NbM) may be profound. NFTs are numerous in the NbM starting early in the disease (Figure 13), and NFTs density parallels cognitive decline severity as does cerebral cortical cholinergic tone. The ARAS neurons supply neurotransmitters that are absent in the intrinsic cortical neurons: the NbM is a major source of cholinergic neurotransmission in the cerebral cortex, which, itself, has no cholinergic cells. NFTs are also thought to affect adversely other ARAS neurotransmitters including histamine, norepinephrine, dopamine, and serotonin (corresponding to neurodegeneration and NFT-containing cells of the tuberomammillary nucleus, locus caeruleus, ventral tegmental area, and pontine raphe, respectively) [169].

In the limbic system, the amygdala has a foundational role because it affectively charges cues, especially in aggressive, fearful, and sexual domains, so that explicit or implicit memory events of a specific anoetic significance can be successfully searched within the appropriate neural nets and, therefore, re-activated [122,171,172]. Emotionally stressful life events impact the amygdala and the hippocampus—areas with a rich density of glucocorticoid receptors—so as to consolidate especially stressful memories. The Papez circuit and the basolateral limbic loop (mediodorsal nucleus of the thalamus, subcallosal area, amygdala, and interconnecting fiber) are also involved. The Papez and the basolateral limbic circuits represent a group of “bottleneck structures” that are interconnected and of high relevance for the extraction of affective, somatosensorial, and social significance of new incoming information [167]. In contrast, when damage involves just the semantic memory system, especially of the hippocampus, patients may forget personal facts and beliefs—where and when they were born, details of their physical appearance, but their anoetic self remains relatively intact [136].

A brief description of the retrogenetic memory impairment in MaNCDs is provided in Figure 9. A complete description of the retrogenesis of memory is detailed in GDS (https://www.alzinfo.org/understand-alzheimers/clinical-stages-of-alzheimers/, accessed on 19 November 2024) [28] and CDR, stages 0 to 3 (https://knightadrc.wustl.edu/professionals-clinicians/cdr-dementia-staging-instrument, accessed on 20 November 2024) [32], while the stages 4 (profound) and 5 (terminal) are as follows [33]:

4—profound: patients’ speech is usually unintelligible or irrelevant; patients are unable to follow simple instructions or comprehend commands; patients only occasionally recognize their spouse or caregiver; patients use fingers more than utensils or require much assistance to eat; patients are frequently incontinent; patients are usually chair-bound; patients are rarely out of their residence; patients’ limb movements are often purposeless;

5—terminal: no comprehension or recognition, patients need to be fed or have tube feedings and are totally incontinent and bedridden.

As regards the non-pharmacological treatment of the mild and moderate stages of MaNCDs, cognitive stimulation is a valid resource [173,174].

Following the retrogenesis of memory, cognitive stimulation is adapted to the impaired handling and writing abilities and to the difficulty to cope of PwMaNCDs until they are entirely lost, usually in the severe stage (CDR 3).

#### 3.4.2. The Socio-Psychological Domain

The progressive impairment of the limbic system, particularly the disconnection from the frontal lobes and amygdala, is responsible for the continuum of the progressive deterioration of social cognition due to compromised critical abilities and judgment and the emergence of infantile uncontrolled behaviors [155].

Before examining the manifestations of retrogenesis in the individual niche formed by adult social cognition, some caveats require attention. The caveats regard the biological difference between the retrieval of infantile uncontrolled behaviors in PwMaNCDs and the infant/child:

1. A PwMaNCD weighs, usually, more than 50 Kg and has the strength of an adult;
2. The long-life developments and storage of millions of events, images, fantasies, thoughts, and behaviors that fill the empty bookcases of the infant mind, building the adult’s EAM, progressively overflow out of the control from the AuMC, impacting, often disastrously, on the psychic sphere of the PwMaNCDs, progressively deprived of critical abilities/judgment and management;
3. The gender may condition the behavior and manifestations in PwMaNCDs: for example, females are more prone to depression, while males are more prone to sexual disinhibition [175,176].

The retrogenetic manifestations and symptoms of the psychological domain are usually assessed with the Neuropsychiatric Inventory (NPI) [41].

**Figure 14:**
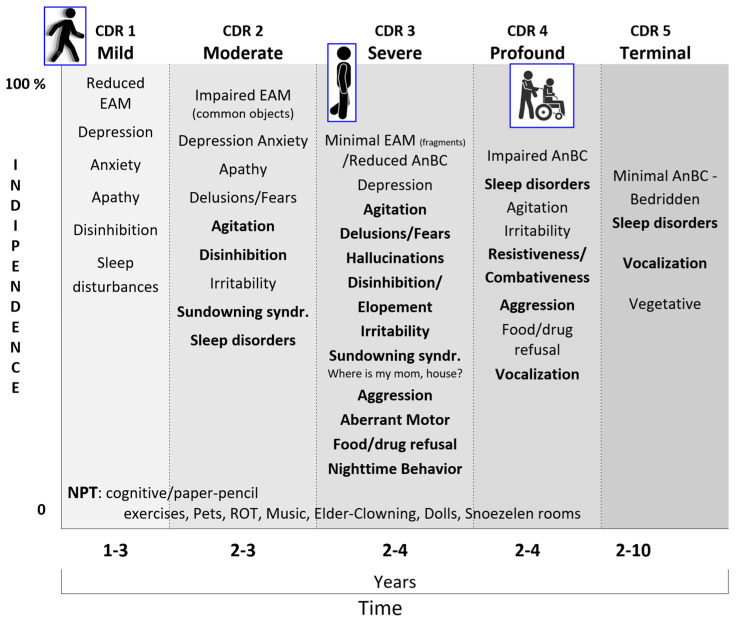
A synopsis of the retrogenetic behavioral manifestations and symptoms of the neurocognitive disorder in the different stages of CDR (stages 0-normal and 0,5-questionable were not included). The symptoms highlighted in bold are the most stressful for caregivers frequently responsible for the placement of the patients in long-term care facilities. AnBC: Anoetic Body Consciousness; CDR: Clinical Dementia Rating extended; EAM: Episodic Autobiographical Memory; NPT: Non-pharmacological Therapy; ROT: Reality Orientation Therapy.

The retrogenetic behavioral disorders affect PwMaNCDs from the mild stage (CDR 1) and increase along the moderate stage (CDR 2), reaching a peak, as severity, daily frequency, and intensity, in the severe stage (CDR 3) (Figure 14) [177,178,179]. As demonstrated before, these disorders appear to be in a reversed order relative to the development of the limbic system, starting with the decoupling of the amygdala in CDR 1 (i.e., depression), then of the NbM and prefrontal cortex (i.e., fears, delusions) in CDR 2, and, finally, of the SCN and HTPAG axis in CDR 3 (i.e., sundowning syndrome, wandering, aggression, disruption of the biological clock and circadian rhythm, and so on) [180,181].

The hallucinations are one hallmark of the retrogenetic psychological symptoms, frequent in the severe stage, that may be reconducted to the child mind prone to believe in fantasies on which the fairy tales, comics, toys, movies, and feasts rely (i.e., the Disney world, flying witches, Santa Klaus flying with the reindeers…). These images and fantasies are progressively controlled by the frontal development of critical abilities and judgement that permits the adult to create an organized mind control and, when woken, a voluntary management of images and fantasies separating them from reality. Examples of the organized adult fantasies may be better understood with old examples like the pharaohs’ Egyptian religion, the masks of the Greek tragedies, Homeric, Dantesque, and medieval poems, and all the ancient and old beliefs that the progress has disavowed. The difference with infant/child fantasies is that, in PwMaNCDs, the voluntary fences of mind control of adult memories are disrupted, and the fantasies, images, and sounds float decoupled from the EAM/AuMC.

The CDR 3 may be considered the transitional stage from the last fragments of EAM/AuMC to the resurfacing of AnBC from the “basement” of infancy. A well-detailed list of the retrogenetic symptoms in this stage is provided by the Nursing Home Behavior Problem Scale [111].

The definitive loss of EAM/AuMC and the transition to AnBC (CDR 3 versus CDR 4) may be empirically recognized when PwMaNCDs lose definitely the mothers’ name [182], even if, for some time, they will continue to search for “my mom” and “my house” not corresponding to the real memories but to the ontogenetic infant/pup survival fears to be left alone and to need a nest.

Moreover, parallelly to the permanent amnesia of the mother’s name, the entrance in the AnBC phase (CDR 4) is usually coupled with progressive amnesia with respect to walking that begins with the impairment of balance and gait and with related falls, often traumatic, until the total loss of walking abilities and the permanent use of a wheelchair. The CDR 4 may be initially characterized by disinhibition, resistiveness, combativeness, and aggression. Then, there is a reduction and a change in behavioral disturbances that are more related to the violation of the AnBC for hygiene, dressing, management of incontinence, and feeding. In CDR 4, PwMaNCDs do not recognize the semantic meaning of the meals, which become “things” no more recognized as foods, especially in the absence of or upon a reduction in the stimuli of thirst and hunger, when the mouth is forced with unsweet “things”. As a rule, the infant is mainly prone to sweet and creamy “things” and frequently reacts against unsweet and grainy “things” during the weaning. Similarly to infants, PwMaNCDs may become uncooperative and/or refuse to feed, feeling these actions as a forced introduction in the mouth of unsweet and grainy “things”, thus miming the conflictual period of the weaning like an oppositional defiant disorder but with the strength of an adult, not of an infant/child [183]. During the course of CDR 4, the behavioral symptoms usually attenuate and mutate progressively into sleep disorders and, sometimes, permanent disruptive vocalization. The transition from CDR 4 to the bedridden CDR 5 stage is marked by the inability to maintain the control of the trunk when seated that is reached by the infant 9 months after birth. In the CDR 5, the behavioral symptoms are sometimes sleep disorders and vocalization that usually tend to cease.

**Figure 15:**
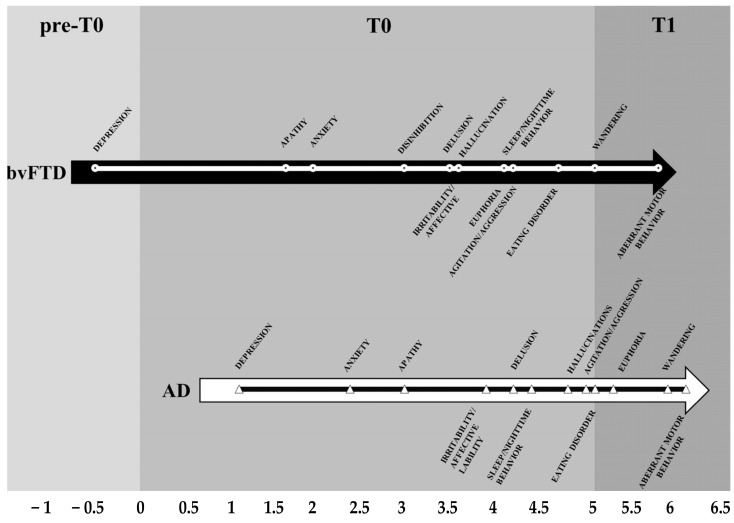
Types and trajectories of the behavioral symptoms in three stages of two different NCDs (bvFTD and AD): preclinical (pre-T0), from the onset to 5 years (T0), and after 5 years (T1). Reproduced with permission from [181]. bvFTD: behavioral variant Frontotemporal Dementia; AD: Alzheimer’s Disease.

There are many aspects that support the retrogenesis of the limbic system as the cause of the socio-behavioral manifestations of the NCDs (Figure 15) [181]:

1. The time of the onset is different depending on the diverse etiologies of the NCDs, but the types and trajectories of the symptoms appear along an overlapping pattern;
2. The overlapping pattern of socio-behavioral manifestations may be embodied in a time neurobiological algorithm:
  - I preclinical—3 years: symptoms are related, originally, to the involvement of the prefrontal–basal-forebrain–amygdala–HTPAG axis [184] circuit in mood alteration (depression, apathy, anxiety, euphoria) and then, after some years, the temperament alteration (irritability, disinhibition, agitation, aggression);
  - II 3–5 years: the worsening of the prefrontal–basal-forebrain–amygdala–HTPAG axis circuit implies symptoms related to the progressive loss of control over the sensorial and semantic inputs (fears, delusion, hallucination) [110], while the detachment of the molecular clock and of the neurovisceral inputs of hungry and thirsty implies the disruption of the circadian rhythm for sleeping and eating;
  - III > 5 years: the complete detachment of the prefrontal–basal-forebrain–amygdala–HTPAG axis circuit from the lower hippocampal circuit implies the resurfacing, from the “basement” of AnBC, of the aberrant motor behavior/wandering of the child, followed by the loss of gait and balance of the infant, and then by the chair-bounded period until the bedridden–vegetative state after the birth stage;
3. The stressful and disabling socio-behavioral manifestations like agitation and sleep and eating disorders affect about 40–50% [43] of PwMaNCDs, but not all the PwMaNCDs as expected if they were entirely provoked by MaNCDs [185]. This aspect is likely due to the resurfacing of the infant’s original temperament in PwMaNCDs which is frequently quite different from the adult acquired behavior, as reported by the caregivers, both in males and females. It is a common experience that infant/child temperament expresses itself like a Gaussian curve whose extremes are: always calm and collaborative and always agitated and oppositive. Therefore, it may be reasonable to presume that PwMaNCDs with a “benign” calm and collaborative behavior or a “disruptive” behavior may recover their original infant/child’s one;
4. The non-pharmacological therapies for the socio-behavioral manifestations of NCDs are applied following the decreasing level of cognitive functioning of PwMaNCDs. In the mild and moderate stages, it is possible to supply the stimulation of cognition, abilities, and games (reality orientation therapy, exercises, drawings, music therapy, …) [173,186,187], while, in the severe and profound stages, mainly games (dolls/pet therapy, elder-clowning, …) [188,189] and sensory memory stimuli (Snoezelen rooms) [190,191,192,193,194]. This pattern is the opposite of the infant/child development that starts with the sensory memory stimuli and games before acquiring the abilities (Figure 15).

#### 3.4.3. The Socio-Functional Domain

**Figure 16:**
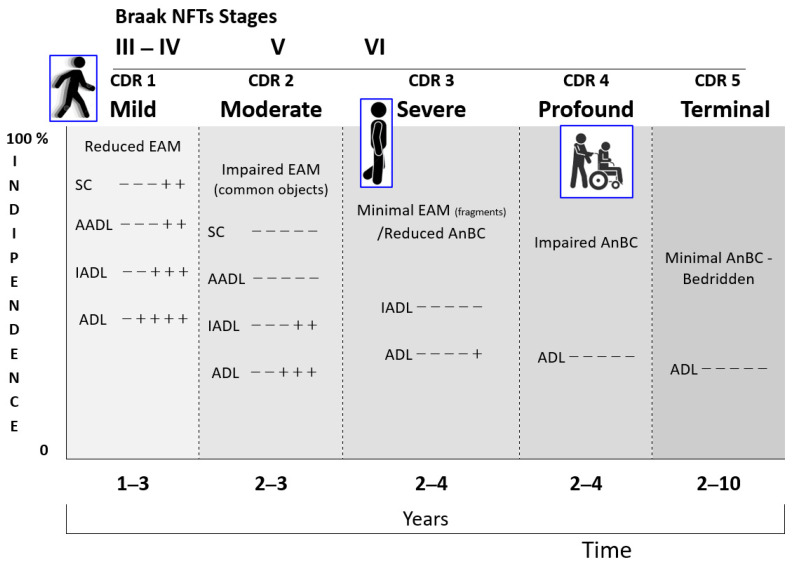
A synopsis of the retrogenesis of socio-functional domains of the neurocognitive disorder in the different stages of CDR (stages 0-normal and 0,5-questionable were not included). Usually, the patients maintain abilities of feeding in the severe stage (CDR 3: ADL − − − − +) that will be lost in the profound stage (CDR 4). SC: Social Cognition (CDR: judgment and problem solving); AADL: Advanced Activities of Daily Living (CDR: community affairs); IADL: Instrumental Activities of Daily Living (CDR: home and hobbies); ADL: Activities of Daily Living (CDR: personal care).

The retrogenetic impairment of the socio-functional domain is examined crossing the NCD neuropathological staging protocol for NFTs/NTs proposed by Braak [154] with the CDR. The NFT stages III and IV correspond to the CDR 1 (Figure 16). In these stages, the lesions reach the hippocampal formation (NFT stage III) and neocortical areas of the basal temporal lobe that adjoin the transentorhinal region laterally. Thereafter, the process encroaches upon the anterior cingulate, insular, and more distant neocortical destinations of the basal temporal lobe (NFT stage IV). Clinically, PwMaNCDs have difficulties solving simple arithmetical or abstract problems, short-term memory or recall deficits, and shows changes affecting social cognition, community affairs, home and hobbies, and need prompting for personal care [155,161].

The NFT stages V and VI correspond to CDR 2 and 3 (Figure 16). The tau pathology spreads superolaterally (NFT stage V) and, finally, reaches the primary neocortical motor and sensory fields, devasting the neocortex (NFT stage VI) [157].

Functionally, the final NFT stages V and VI correspond to a fully developed AD, where PwMaNCDs become totally incontinent, cannot dress themselves unaided or recognize persons once familiar to them (spouse, children). With the passage of time, they can no longer walk, sit up, or hold up their head unassisted. Increasing rigidity of the large joints leads to irreversible contractures of the extremities and to immobility. Primitive reflexes, normally seen only in infants, also reappear in the terminal stage of MaNCDs [10].

## 4. Discussion

This description of the infant anoesis (AnBC) and its transition to the adult autonoesis (EAM-AuMC) in the light of the individual biosphere adds a contribution from a multidimensional person-centered point of view to the Reisberg’s retrogenesis system of the progression of MaNCDs.

The AnBC is strongly connected to raw sensorial and perceptual abilities, various subcortical affective processes, and intrinsic affective value structures that take place into the limbic and paralimbic structures.

The emergence of EAM/AuMC in childhood is coupled with the progressive stabilization of the biological clock that regulates the circadian rhythms for sleep–wake, hormone production, and, importantly, memory.

The EAM is “…a past- and future-oriented, context embedded neurocognitive memory system that receives and stores information about temporally dated episodes or events, and temporal-spatial relations among them from one’s past” [137,138].

The EAM differs from other forms of memory because requires an extended sense of self that engages in mental time travel.

Time awareness and orienting are related especially to the prefrontal cortex, while the proper perception of time and time epochs seems to engage the parietal cortex as well as the diencephalic structures.

When considering the BPS and functional retrogenetic cascade, some considerations and details may be of interest for a multidimensional, person-centered model.

The development of cognition and knowledge from birth to adulthood is due to the progressive two-stage maturation of the whole limbic system: firstly, the infant AnBC and then the adult EAM/AuMC, with AnBC embedded in EAM/AuMC as a continuum.

The genetic absence of EAM/AuMC in infancy is the key point that permits the acquisition of the anoetic but voluntary body control and consciousness by every newborn.

The growing functioning of EAM/AuMC in the childhood–adolescence period is the basis for the development of the “self” after the AnBC and not together.

In the retrogenesis, the impairment of the cognitive, psycho-behavioral, and functional symptoms of the MaNCDs is progressive and interconnected because expression of the reversed progressive impairment of the individual adult biosphere.

The overlapping of the cohort of cognitive, psycho-behavioral, and functional symptoms of MaNCDs is substantially independent from the etiopathogenesis and due to a retrogenetic impairment of the limbic system.

The actual etiopathogenesis of MaNCDs, correlated prevalently with the damage of the hippocampus, is restrictive and inadequate to explain the galaxy of overlapping manifestations and symptoms of MaNCDs.

Many neuropathologic studies have demonstrated the involvement of the amygdala, basal forebrain nuclei, and frontal cortex as responsible for the retrogenetic psychological and behavioral symptoms of MaNCDs (apathy, depression, anxiety), frequently anticipatory of the amnesic symptoms.

This remark outlines the necessity to treat, always, the depression to prevent MaNCDs [195].

Moreover, the involvement of the suprachiasmatic nuclei (the molecular clock) and of the HTPAG axis is responsible for sleeping and eating disorders, frequently concomitant with the amnesic symptoms.

The long-term body memories acquired with the AnBC remain stocked for life in a “basement” fundamental nucleus over which progressively develops the continuum of the intertwined blocks of the EAM/AuMC. In the BPS retrogenesis, the EAM/AuMC vanishes along the mild, moderate, and severe stages of MaNCDs and remains only the “basement” AnBC that vanishes too, losing progressively the ADL in a retrogenetic order of acquisition where the last function to be deleted is chewing and swallowing.

The eating, together with chewing, disorders of the advanced stages of MaNCDs are solved with the infant creamy sweet foods until the swallowing memory is evocable from the AnBC of PwMaNCDs.

Limitations. The ultimate reason of this work lies in the effort to implement the BPS retrogenetic knowledge of NCDs in the frontline of caring for PwMaNCDs, especially caregivers, nurses, general practitioners, psychologists, and social workers. Actually, even if the multidimensional, person-centered model is based on cross-professional tools, validated from a long time ago (retrogenesis, staging, BPS approach, comprehensive geriatric assessment), different limitations hinder the applicability:

- Cultural: the lack of an interdisciplinary consensus among physicians, general practitioners, specialists, caregiver associations, nurses, psychologists, and social workers to consolidate definitively the model into a unique system of “science of NCD management” [6];
- Educational: the absence of different educational levels and programs targeted at the different actors of the care and cure of PwMaNCDs; for example, the synopsis in Figure 14, Figure 15 and Figure 16 is a visual proposal to introduce MaNCDs management.
- Social: the presence of increasing and pervasive stigma toward PwMaNCDs, involving all the potential actors of caring and interesting the knowledge of dementia, attitudes and beliefs, and behaviors: 80% of the general public and 65% of health and care professionals think that NCDs are a normal part of ageing [196].
- Structural: the availability of interdisciplinary teams (physician, nurse, nurses’ aid, caregiver, physiotherapist, psychologist, social worker, volunteers, and others) in the different settings where PwMaNCDs live or to which they are transferred (home, long-term care facility, nursing home, hospital, hospice, and other).

## 5. Conclusions

The multidimensional comprehensive geriatric assessment may represent a core that unifies the evaluation of PwMaNCDs in their BPS and functional retrogenesis as a global continuum, not limited only to the illness.

This “science of NCD management” addresses many relevant options for future research, particularly:

(1) The quick diagnosis of symptoms related to the early involvement of the limbic system (mood and/or metamemory disorders) firstly in the general practice, as it has been undoubtedly demonstrated that, in AD, the deposition of tau and beta amyloid proteins in the brain begins many years before; for this purpose, the future availability of specific blood biomarkers should ameliorate the frequent diagnostic delay;
(2) The development of new non-pharmacological strategies and pharmacological therapies primarily targeted at the involvement of the limbic system in the impairment of social cognition and the emergence of infantile uncontrolled behaviors in strong adults with MaNCDs;
(3) The implementation of web technologies to ensure the needed online care, cure, rehabilitation, and support to PwMaNCDs.

## Figures and Tables

**Table 1:** The basic functions of body independence.

Functions	Inborn	Learned
1. Swallowing solids		+
2. Language	+	
3. Napping/sleep	+	
4. Nictemeral sleep		+
5. Balance		+
6. Gait, walk, run		+
7. Sphincteric control		+
8. Dressing		+
9. Hygiene		+

**Table 2:** The four core affect categories of anoetic consciousness in the primal systems. The affects in bold are the most relevant, adapted from [64,99].

ISTINCTS AND AFFECTS						
ISTINCTIVE	EMOTIONAL	SENSORY	HOMEOSTATIC			
**self-preservation (survival)**	**crying-smiling**	**pleasure**	**air**	independent	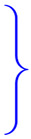	Experiences and Behaviors—AnBC
creativity	fear	**sweet**	**thirst**	maternal bond		
curiosity	play	**grainy**	**hunger**	maternal bond		
passions	seeking	**pain**	light	maternal bond		
wills	lust	salty	cold	maternal bond		
	rage/anger	sharp	heat	maternal bond		
	care	noise				
	panic	quietness				

AnBC: Anoetic Body Consciousness.

**Table 3:** The infantile emotional affects and their neuroscientific sense, adapted from [99].

(1) Crying/smiling: the genetic indistinct on–off primitive system of communication that comprises vocalization as a first sign of developing language or homeostatic wellbeing. (2) Fear: it defends the organism from existential threats. (3) Play: together with seeking, the play system teaches the infant to interact with the unknown surrounding world to progressively know it. (4) Seeking: together with play, it is essential to discover the territory for finding everything one needs to survive. (5) Lust: it is heavily connected to the seeking system, the primary requirement for perpetuating the species, and is considered central to replication. (6) Anger: it is a response to fear or frustration, when access to desired resources is prevented. (7) Care: tied with lust, care supports growth and maturation through parental devotions. It is stronger in women than in men, in whom it is also present.

**Table 4:** Manifestations and symptoms related to damage of regions of the limbic system [167].

	MTL	D	BF
Capabilities of conscious reflection and self-awareness	+	+/−	−/+
Difficulties with time relationships	−	−/+	−
Problems with attention and concentration	−	−	+
Unbalanced impairment of recall as opposed to recognition	−	−	+
Emotional instability and mood disorders	−	−/+	+
Impairment in executive functions	−	+/−	+
Manifestations of disinhibition and perseveration	−	+/−	−/+
Anosognosia	−	−/+	+
Confabulation	−	−/+	+

MTL: Medial Temporal Lobe, D: Diencephalon, BF: Basal Forebrain.

## Abbreviations

AD	Alzheimer’s Disease
ADL	Activities of Daily Living
AnBC	Anoetic Body Consciousness
ARAS	Ascending Reticular Activating System
AuMC	Autonoetic Mind Consciousness
BPSD	Behavioral and Psychiatric Symptoms of Dementia
BPS	Biopsychosocial (pathway)
bvFTD	behavioral variant of Frontotemporal Dementia
Braaks NFTs-ST	Braaks NFT Staging Tool
CDR	Clinical Dementia Rating scale
CNS	Central Nervous System
DAEs	Developmental Age Equivalents
EAM	Episodic Autobiographical Memory
EN	Enteral Nutrition
FAST	Functional Assessment Staging Tool
FTD	Frontotemporal Dementia
FOF	Fear of Falling
GDS	Global Deterioration Scale
HPTAG axis	Hypothalamic–Pituitary–Adrenal-Gonadal axis
IA	Infantile Amnesia
IADL	Instrumental Activities of Daily Living
Major NCD	MaNCD
NbM	Nucleus basalis of Meynert
NCD	Neurocognitive Disorder
NFTs	Neurofibrillary Tangles
NPI	Neuropsychiatric Inventory
NTs	Neuropil Threads
PN	Parenteral Nutrition
PwMaNCD	Person affected by a Major Neurocognitive Disorder
ROT	Reality Orientation Therapy
RSN	Resting-State Networks
SCN	Suprachiasmatic Nuclei
